# Biosensors for the Detection of Circulating Tumour Cells

**DOI:** 10.3390/s140304856

**Published:** 2014-03-10

**Authors:** Clotilde Costa, Miguel Abal, Rafael López-López, Laura Muinelo-Romay

**Affiliations:** 1 Translational Medical Oncology, Health Research Institute of Santiago (IDIS); Complexo Hospitalario Universitario de Santiago de Compostela (SERGAS); Trav. Choupana s/n 15706 Santiago de Compostela, Spain; E-Mails: miguel.abal.posada@sergas.es (M.A.); Rafael.lopez.lopez@sergas.es (R.L.-L.); 2 Unity of CTCs analysis Translational Medical Oncology, Health Research Institute of Santiago (IDIS); Complexo Hospitalario Universitario de Santiago de Compostela (SERGAS); Trav. Choupana s/n 15706 Santiago de Compostela, Spain; E-Mail: Laura.muinelo.romay@sergas.es

**Keywords:** circulating tumour cells (CTCs), isolation, enrichment, detection, sensors

## Abstract

Metastasis is the cause of most cancer deaths. Circulating tumour cells (CTCs) are cells released from the primary tumour into the bloodstream that are considered the main promoters of metastasis. Therefore, these cells are targets for understanding tumour biology and improving clinical management of the disease. Several techniques have emerged in recent years to isolate, detect, and characterise CTCs. As CTCs are a rare event, their study requires multidisciplinary considerations of both biological and physical properties. In addition, as isolation of viable cells may give further insights into metastatic development, cell recovery must be done with minimal cell damage. The ideal system for CTCs analysis must include maximum efficiency of detection in real time. In this sense, new approaches used to enrich CTCs from clinical samples have provided an important improvement in cell recovery. However, this progress should be accompanied by more efficient strategies of cell quantification. A range of biosensor platforms are being introduced into the technology for CTCs quantification with promising results. This review provides an update on recent progress in CTCs identification using different approaches based on sensor signaling.

## 1. Introduction

Early dissemination of tumour cells is usually undetectable in patients by conventional histopathology examination. Recently, immunocytochemical and molecular assays have been developed for the specific detection of metastatic tumour cells in lymph nodes, peripheral blood, or bone marrow, prior to the manifestation of metastasis [1–7].

In addition to the clinical relevance inherent to the understanding of the process of metastasis, early detection of circulating tumour cells (CTCs) could be useful for the identification of patients who require additional systemic therapies after resection of the primary tumour. Although the aim of these therapies is the prevention of metastasis, the selection of patients who could benefit from the treatment is nowadays mainly based on the statistical risk of recurrence. In fact, the role of CTCs as a prognosis factor and their use for early detection of metastasis events are recognised for several tumours such as breast, colorectal, lung and prostate cancers [8–12].

Inclusion of the sequential follow-up of CTCs in clinical trials could provide information in the early stages about the therapeutic efficacy of the drugs against the presence of metastatic tumour cells. Likewise, elimination of these CTCs could represent an intermediate endpoint in clinical trials with antitumour drugs [13].

To enrich or sort CTCs from peripheral blood, several approaches have been published, some of which are summarised in this review. CTCs enrichment methods are based on physical or biological cell properties such as size or specific marker expression. Although most of the CTCs isolation techniques can be carried out in a semi-automated manner, they are laborious procedures with variable efficiency [14]. Besides, after the isolation process, a next step is required to identify CTCs with high specificity. This step is normally tedious and time-consuming, using techniques such as PCR, immunofluorescence, or flow cytometry [15]. To solve these limitations, detection approaches using sensor technology are being combined with traditional CTCs isolation procedures.

Biosensors are defined as analytical devices composed of a recognition element of biological origin integrated into or associated with a physico-chemical transducer. The biological event produces a measurable change in a solution property, which the transducer converts into a quantifiable electrical signal [16]. Often, the term biosensor is used when the concentration of substances or other biological parameters are determined even where a biological recognition element is not used directly.

Recognition elements include enzymes, immunoagents, DNA segments, and even whole cells, all of which are coupled to different modes of transduction [17]. The mode of transduction covers several approaches, including electrochemical, optical, and mass measurement. Some biosensors are considered point-of-care devices (POC). POC diagnostics are medical tools that can be used outside of a hospital setting and tend to be portable, fast, and relatively inexpensive [18]. More advantages of biosensor application are that they are easy to use, miniaturised (lab-on-a-chip devices), and offer robust results compared with classical analytical techniques such as immunohistochemistry or ELISA [19].

In the last years, several label-free biosensing technologies for the detection and monitoring of clinical relevant molecules such as glucose, hCG or cardiac markers for diabetes, pregnancy test, or cardiac diseases have been reported [20,21]. Currently, the use of biosensors for live CTCs detection and monitoring is a challenge, taking into account the great biological heterogeneity of CTCs. This review provides an update on recent progress in CTCs identification using different approaches based on sensor signalling.

## 2. Techniques for CTCs Isolation

Isolation of CTCs from whole blood could be done using biological (antigen expression) or physical features. Here, we summarise some of the most used methods for CTCs isolation from whole blood (Table 1).

### 2.1. Immunoaffinity/Immunobinding

Antibodies are extensively used to functionalise magnetic beads or nanostructured substrates (silicon nano/micropillars) that could be used to separate CTCs from blood cells [29,40,41,53–55]. This approach is limited by antibody-antigen specificity and the complete process needs long interaction times. The antigen mostly used is EpCAM, an epithelial marker overexpressed in some carcinomas [22,23,40,56]. CTCs undergo changes in their epithelial signature during the metastatic process, avoiding the use of EpCAM as a universal marker [57–59]. Therefore, all efforts are focused on characterisation and identification of additional markers able to distinguish CTCs from their counterparts in blood.

Currently, CellSearch^®^ (Veridex LLC, now Jenssen Diagnostics LLC, Raritan, NJ, USA) is the only technology approved by the US Food and Drug Administration for CTCs quantification in metastatic breast, prostate, and colon cancers. This technology uses magnetic beads coated with an anti-EpCAM antibody for CTCs isolation and the identification is mainly based on cytokeratins expression [8,9,24]. Although CellSearch^®^ is an accepted platform with high value for cancer prognosis and monitoring, the low purity of the CTC-enriched samples, its low sensitivity, and its limitation to some cancer types [24] reinforce the need for more effective technologies for CTCs analysis.

In this sense, the combination of EpCAM-based CTCs immunoisolation with PCR quantitation methods represents an alternative to improve detection rates. Using this strategy, our group, among others, has obtained good results for the detection and characterisation of CTCs from metastatic colorectal cancer (mCRC) [60,61].

This year, another Spanish group reported a novel system for CTCs counting in an *in vitro* model, using ImageStream (Amnis, Seattle, WA, USA). ImageStream is an imaging cytometry device that combines flow cytometry to select EpCAM-expressing cells and subsequent identification by fluorescence microscopy. The group compared this method with the CellSearch^®^ system [27] and found an expected ratio of CTCs in most cancer patients [25]. The use of flow cytometry *in vivo* was tested several years ago [62–64]. This technology is now combined with current ones to achieve better results.

Recently, an immune-filtration approach has also been developed and isolation of CTCs from lung cancer patients has been shown [29]. This device adds a magnetic sifter that has been re-engineered from a previous sifter [30]. Cells are isolated by immunoaffinity using magnetic nanoparticles bound to anti-EpCAM antibodies. Then, cells can be imaged directly on the magnetic sifter array and harvested by moving out the field and washing. Improvements include higher efficiency in capture with better throughput, rapid imaging of captured cells, and harvesting of viable cells, avoiding the loss of cells in preparatory steps, compared with other methods.

Importantly, a portable device (CellCollector™, GILUPI NanoMedizin, Potsdam, Germany) based on EpCAM expression has been developed in the last years. This medical system showed high specificity and sensitivity for isolation of CTCs *in vivo* from circulating peripheral blood of breast cancer or non-small cell lung cancer (NSCLC) patients. The system is inserted through a standard venous cannula into the cubital vein for 30 min. After the enrichment step, CTCs are identified by EpCAM and/or cytokeratin expression [31]. This device is considered a promising tool for monitoring the course of the cancer disease and the efficacy of anticancer treatment *in vivo*.

### 2.2. Physical Properties

CTCs could be also separated from blood cells according to their size. Isolation based on *cell size* has two main advantages: a higher capture efficiency and independence of antigen expression. When epithelia-mesenchymal transition (EMT) takes place as the step previous to metastasis, some epithelial markers are lost [65]. We have recently described the acquisition of a plasticity and stemness phenotype in CTCs from endometrial cancer patients, probably related to their ability to promote distant metastasis [66].

Several platforms using size as the isolation method to detect CTCs in blood were reported in recent years [32,37,67,68]. Example of these commercially available devices are ISET^®^ (Rarecells Diagnostics, Paris, France) and ScreenCell^®^ (Screencell, Westford, MA, USA) [32–34,67]. The disadvantages of these systems are that they provide low CTCs purity, requiring in most cases further enrichment, and the fact that leucocytes could overlap in size with CTCs. Additionally, smaller CTCs or fragments of CTCs could be lost. To avoid classical limitations in CTCs isolation with systems based on cell size, in 2007, Zheng and co-workers described a micro-electro-mechanical system (MEMS) in which cells are immobilised, allowing their direct contact with electrodes. This microfilter device can capture and perform electrolysis and genomic analysis of human CTCs in not much time [37]. The described filters were improved, allowing isolation of live cells [38].

There are also methods for CTCs isolation based on the differences in density between epithelial and blood cells. Density centrifugation methods separate erythrocytes, platelets, and polymorphonuclear cells in the pellet; mononuclear cells (MNCs, including tumour cells) are collected in the interphase. One of the commercialised methods based on the density centrifugation system is OncoQuick^®^ (Greiner Bio-One, Frickenhausen, Germany) which achieves better results than the current standard method with Ficoll™ (GE Healthcare, Pittsburgh, PA, USA) [39].

*Microfluidics* has been demonstrated to be viable platforms for CTCs analyses that can be integrated into other processing steps to fully automate sample processing. These platforms could combine both physical and biological cell isolation approaches [40,41]. Microfluidic devices using affinity selection typically demonstrate higher purity compared with size-based selection, but at the expense of throughput [40,42,69–71]. In 2007, Nagrath reported their innovative development of the ‘CTC-chip’. This platform isolates viable CTCs by affinity to anti-EpCAM-coated microspots under controlled laminar flow conditions. The device demonstrated higher sensitivity, selectivity, and yield compared with techniques based only on immunomagnetic beads, including CellSearch^®^ [25,40,72].

The efficacy of the CTC-chip has been proved for metastatic lung, prostate, pancreatic, breast, prostate, colon [40], and non-small cell lung cancers [73,74]. Other options based on the CTC-chip appeared as herringbone channels, which obtained higher recovery at the expense of low purity [27]. There are some variants such as silicon nanopillars [43] or sinusoidal channels to increase throughput [42].

In 2009, another device based on microfluidics was reported [44]. This label-free microdevice considers physical properties like deformability and size, as CTCs are generally larger and stiffer than blood cells. This approach is able to isolate viable cancer cells from blood of lung, breast, and colon cancer patients [44]. Optimisation by computational analysis enhances isolation efficiency [45].

Other microfluidic devices for CTCs separation combine hydrodynamic flow with size-based separation [46–48]. The main difference is the subsequent step: application of dielectrophoretic forces through the microelectrode arrays onto the field. However, CTCs isolation using dielectrophoresis presents limitations in time, sample volume, and cell loss with the added density gradient step [49–51]. Another study was designed based on the Moon data [52]. The parallel multi-orifice flow fractionation device (MOFF) is composed of four single MOFF channels to improve throughput based on hydrodynamic separation.

## 3. Biosensors for CTCs Quantification

Several biosensing technologies have been developed for CTCs detection and monitoring. Biosensors are composed of different parts: bioreceptors, an electrochemically active interface where specific biological events take place, giving rise to a signal; a transducer that translates biochemical reaction into electrical signal and amplifies it; a signal processor (software); and an interface to show data to the operator [17]. Biosensors may be classified according to the biological specificity-conferring mechanism or the mode of physicochemical signal transduction [75]. Taking into account the signal transduction, we could categorise biosensors into electrochemical, mass change or optical [76,77].

A potential application of biosensors in the field of CTCs is the possibility to provide real-time and highly efficient quantification, improving on the CTCs analysis strategies based on direct immunohistochemical or indirect PCR identification. Below, we summarise the biosensors being applied in the oncology area to detect CTCs, taking into account both the nature of the recognition and the transduction signal (Table 2). Regarding recognition elements, aptasensors have special utility for CTCs counting devices.

### 3.1. Aptasensors

Aptasensors use aptamers as a recognition element [86]. Aptamers are artificial nucleic acid (DNA or RNA) ligands that can be selected from combinatorial libraries of synthetic nucleic acids. They have different and specific binding characteristics to their targets. Using the systematic evolution of ligands (SELEX) process, aptamers can be isolated from randomly synthesised RNA or DNA pools. Numerous high-affinity aptamers have been used against a wide variety of target molecules and also whole cells [87,88]. Aptamer approaches are superior to those with antibodies because they can be selected against non-immunogenic and toxic targets [86].

Taking into account the difficulty of direct detection of CTCs (one CTC/10^6^ MNCs) and the presence of ∼10^3^ to 10^4^ copies of target RNA per CTC, several aptasensors have been developed for CTCs evaluation. Recently, Zhang *et al.* described a microfluidic bead-based nucleic acid sensor for sensitive CTCs detection in blood samples using multienzyme-nanoparticle amplification and quantum dot labels. In this technology, functionalised gold nanoparticle (AuNP) probes are hybridised with the target DNA molecules that are also coated with the functionalised microbeads. Once the catalytic reaction by horseradish peroxidase (HRP) occurs, streptavidin-conjugated quantum dots bind to the introduced biotin moieties on the surface of microbeads to emit fluorescence. Due to the dual signal amplification strategy, the developed microfluidic bead-based nucleic acid sensor could discriminate as low as 5 fM of synthesised carcinoembryonic antigen (CEA) gene fragments. Interestingly, using spiked colorectal cancer cell lines HT29 in the blood, the detection limit of this chip-based approach was found to be 1 HT29/1 mL blood sample. Therefore, this device provides a high-sensitivity method for CTCs analysis and monitoring [78].

Interestingly, antisense oligonucleotides (ASOs) were used together with resonance frequency shifts induced by AuNPs to form a platform for CTCs identification. The platform consists of a chip-based device, which utilises ASOs covalently attached to metallic or silica-coated nanowires (NWs) to detect marker RNAs for various cancer types. Target RNAs are bound to the ASO-derivatised NWs by sandwich hybridisation. A second ASO, attached to a single 50 nm AuNP, is hybridised to a different site on the bound target RNA. Two stringent hybridisations increase specificity and NW-resonator (NR) sensitivity. The hybridisation detection is accomplished by an optical approach, by measuring the shift in resonance frequency of the NRs. An optimisation of the platform was performed, using RNA from PCA3 cells as a marker for prostate cancer with high specificity and quantitative sensitivity [79].

### 3.2. Electrochemical Transducers

Electrochemical sensors are based on the detection of current or potential changes caused by interactions at the transducer interface. Antibodies, receptors, or aptamers are recognition elements which, together with solid electrode surfaces (Au, Ag, graphite- or carbon-based conductors, electrode arrays), react to electrical impulses such as potential or current [89,90]. When a redox mediator is included in the solution, the change in the electrochemistry of this species is directly related to the molecular recognition. These electrochemical biosensors have great value for clinical application due to their high sensitivity and customisation, simplicity, and low cost compared with other sensors based on optical transduction. One problem with this strategy is the fact that some target molecules lose their binding properties because of the labelling. To avoid this problem, there is an important effort to develop label-free electrochemical systems [91].

*Amperometry* is the most often employed technique in biosensors for clinical analysis. This technique has the advantage that certain chemical species are oxidized or reduced at inert metal electrodes, motivated at a constant applied potential. Two or three electrodes may form an amperometric cell. One of these electrodes, the working electrode, is frequently composed of a metal such as Ag or Au. The other electrode (reference electrode) is usually made of Ag/AgCl. The potential applied to the working electrode is measured and controlled referring to the other electrode, which has a fixed potential. Sometimes, this device includes a third electrode (auxiliary or counter electrode) [92,93]. This type of electrochemical transduction was employed by Moscovici *et al.* for the development of a novel microfabricated glass chip device that provides rapid detection of circulating prostate cancer cells. The method uses a gold electrode array with tunable sensor surface areas that are coated with anti-EpCAM antibodies for CTCs detection. The binding of a prostate cancer cell onto the antibody-modified gold surface alters the interfacial electron transfer reaction of a redox reporter, and allows high-efficiency readout of small cell populations within 15 min [80].

Another electrochemical technique used for the biosensor development is *potentiometry*. These devices allow the quantification of an electrical potential difference between two electrodes when the cell current is zero. Both electrodes are known as the indicator and reference electrodes. The reference electrode is required to provide a constant half-cell potential. The indicator forms a variable potential, depending on the activity or concentration of a specific compound. The alteration in potential is logarithmically related to the compound concentration [94,95]. For example, the ion-selective electrode (ISE) for the measurement of electrolytes is a potentiometric technique routinely used in clinical chemistry. Potentiometric approaches were also used for cancer cell and biomarker monitoring. To investigate the single cell response to drugs in an *in vitro* assay, Jia and co-workers [96] used a light-addressable potentiometric sensor (LAPS). This system could be adapted for CTCs analysis.

*Impedance* techniques are also included in electrochemical biosensors and have been proved to be a promising method for pathogenic bacteria detection due to their portability, speed, and sensitivity; more importantly, they can be used for on-the-spot detection [97,98]. Electrochemical impedance spectroscopy (EIS) is the most used impedance approach. It describes the response of an electrochemical system (cell) to an applied potential (small amplitude sinusoidal voltage signal). The frequency dependence of this impedance can disclose underlying chemical processes [99].

Kamande *et al.*, as mentioned above, developed a thermoplastic modular microsystem for high-throughput analysis of CTCs directly from whole blood. The system is composed of three functional modules based on electrochemical impedance transduction. The first module consisted of a thermoplastic CTCs selection module designed with Z configuration and composed of high aspect ratio (30 μm × 150 μm) channels. These channels contain anti-EpCAM antibodies. The system allowed scalability in terms of throughput by employing channel numbers ranging from 50 to 320. In addition, an impedance sensor module was used for label-less CTCs counting. Using this sensor, they were able to discriminate between leukocytes and CTCs based on size differences. This is the cell characteristic measured at the operating frequency (40 kHz) that serves as a pre-screening tool before immunocytochemical CTCs staining. The utility of the system was demonstrated using blood samples from patients with both local resectable and metastatic pancreatic ductal adenocarcinomas (PDACs) with high purity (>86%) and yield (mean of 53 CTCs/mL) [81].

Changes in solution conductivity have also been used as a transduction mechanism in enzyme-based biosensors. *Conductometry* is defined as the measure of the ability of ions in solution to carry current between two electrodes. Although clinical application of conductometric biosensors is limited due to the ionic background of clinical samples, this type of signal transduction was also used for CTCs detection.

Adams *et al.* reported a system for CTCs quantification based on microfluidic and highly specific single cell conductivity. The microfluidic device consisted of a series of high aspect ratio microchannels (35 μm width × 150 μm depth) that were replicated in poly(methyl methacrylate), PMMA, from a metal mould master. The microchannel walls were covalently coated with EpCAM antibodies. The CTCs capture efficiency was high (>97%) due to the option of design capture channels with the appropriate widths and heights. Isolated CTCs were released from the surface using trypsin and then enumerated on-device using a novel, label-free solution conductivity route able to detect single tumour cells moving through the detection electrodes. The authors obtained a 100% detection efficiency and good specificity for CTCs due to scaling factors and the suboptimal electrical properties of potential interferences (non-CTC cells) [42]. Besides, a micro-Hall detector developed by Issadore and co-workers [82] uses semiconductor technology as a strategy to enable high-throughput CTCs screening (approximately 10^7^ cells/min). The value of this device was demonstrated in ovarian cancer patients.

Improvement of electrochemical signal has been achieved with the utilisation of different *nanomaterials*, including nanoparticles, nanowires, nanoneedles, nanosheets, nanotubes, *etc*. Nanosized materials have been used with different strategies: direct wiring of enzymes to the electrode surface; to support electrochemical reactions and to amplify signals of a biorecognition event.

Although the use of nanomaterial in the field of oncology is mainly focused on tumour biomarker recognition such as PSA for prostate cancer diagnosis and monitoring [100], this technology was recently applied for CTCs detection. In 2008, Shao *et al.* reported a single nanotube field effect transistor array, functionalised with anti-IGF1R and Her2 antibodies. These biomarkers exhibited high specificity for human breast cancer cells (MCF7 and BT474) in human blood. The device was designed to have small spacing between the electrodes (1 μm) in order to capture a single alive cell (8–12 μm) by specific interaction with their cell surface receptors. The free energy change due to multiple simultaneous cell-antibody binding events exerted stress along the nanotube surface. In consequence, the electrical conductivity decreased due to an increase in band gap that allowed the molecular sensing of these CTCs [83].

In 2009, different Spanish groups from Barcelona, Zaragoza, and Vigo developed a novel cell sensor based on DNA sensors and immunosensors coupled to a new electrocatalytic detection method for AuNPs [101]. This platform allows rapid detection and identification of *in situ* cell proliferation. Specific antibodies were conjugated to AuNPs and their catalytic activity in hydrogen formation made the quantification of attached cells possible. The group used this device for the detection of surface molecules on tumour cells but one of its potential applications could be cell detection in biological fluids.

### 3.3. Mass Change Transducers

This type of transduction is also classified as a label-free technology and offers the option to analyse cell movement (attachment and spreading). This approach provides a high rate and sensitivity of detection, together with real-time results. Quartz crystal microbalance (QCM) and surface acoustic wave (Love wave) systems, two mass change strategies, are employed to monitor the adhesion of animal cells to various surfaces and record the behaviour of cell layers under different conditions. QCM devices, where the specific antibody is immobilised on the sensor chip, have been used for a wide range of applications in the medical field such as cancer marker detection [102–104]. Several devices are now available on the market based on this approach to monitoring cancer evolution through biomarker analysis. One example is the QCMA 1 Sensor instrument (Sierra Sensors GmbH, Hamburg, Germany), which is a fully automated sensor for PSA analysis [104].

Recently, *in vivo* photoacoustic flow cytometry (PAFC) for label-free detection of mouse B16F10 CTCs in melanoma-bearing mice using melanin as an intrinsic marker was described [84]. This platform demonstrated a low level of background signals and favourable safety standards for being implanted in the future for melanoma diagnosis and real-time monitoring of therapy. This system is being evaluated for its clinical application in a clinical trial, which is in the patient recruitment phase (NCT01776905).

### 3.4. Optical Transducers

Optical transducers include fluorescence, interferometry, and spectroscopy of optical waveguides and surface plasmon resonance (SPR). Several commercially available devices use fluorescence labels for detection. In these systems, fluorescence reporters convert the detection of a specific biological parameter into an observable fluorescent signal. A sensitive and specific fluorescence resonance energy transfer (FRET) biosensor was developed by Mizutani and colleagues. They applied it to detect the activity of BCR-ABL kinase in live cells [105]. This biosensor allowed the detection of cancerous and drug-resistant cells, and the evaluation of kinase inhibitor efficacy.

On the other hand, direct optical transducers, such as internal reflectance spectroscopy, SPR, and evanescent wave sensing have attracted high interest because they do not need a label to detect the event, avoiding a separation step to remove the labelling. The application of a label-free microchip biosensing device for the detection of CTCs was described for the first time by Kumeria *et al.* [85]. This approach is based on label-free reflectometric interference spectroscopy (RIfS) on novel nanoporous anodic aluminium oxide (AAO). Biotinylated anti-EpCAM antibodies were covalently attached to the modified AAO surface and used as a CTC-capturing platform. The binding of CTCs to the antibody-modified AAO surface provided a strong influence on the RIfS signal, causing a wavelength shift in the Fabry-Perot interference fringes, which was applied for CTCs detection. For gold-coated AAO functionalisation, self-assembled monolayers (SAMs) of mercaptoundecanoic acid (MUA) with a long carbon chain were selected. These provide the appropriate carboxylic acid terminal group for subsequent streptavidin-biotin antibody attachment and a laxer binding surface for cancer cells, and prevent non-specific cell binding by electrostatic repulsion.

The performance of this device was characterised by using EpCAM expressing/non-expressing cells. The system required an optimal concentration of 1000–100,000 cells/mL in PBS or blood. This device is considered to have high potential for clinical application because its improvement by microfluidic and optical system designs could provide a lower detection limit (<10 cells).

## 4. Future Directions

Despite emergence of several approaches for CTCs analysis in the last decade, currently no optimal platform is available. A low-cost and portable device would be a key tool for decreasing cancer mortality. Currently, there are dozens of clinical trials to validate new devices for CTCs detection some of them based on microfluidics, such as NCT01734915 or NCT01193829. Other ones are in progress to validate CTCs monitoring for prognosis and response to different therapies (for example, NCT01625702 and NCT01658332 using CellSearch^®^ technology). The prediction is that within the next 10 years, CTCs analyses will be regularly done in combination with classical tests to improve the management of cancer patients [106]. In this regard, there is a limitation in the detection of CTCs due it is an extremely rare event. The low frequency of CTCs in blood can give skewed results when only 7.5 mL of blood are analyzed. It would be interesting to implement the CTCs analysis in greater amounts of blood. We also have to focus on minimizing false positives and negatives that may occur due to detection of other cell types in the whole blood. Taking into account that the more aggressive cell subpopulations vary their molecular markers, for a better CTCs detection we should also consider CTCs' morphology in combination with their molecular profile or even in parallel with circulating DNA. It is clear that an improved sensitivity of CTCs isolation and quantification must be achieved, and the combination of standard techniques with biosensors for CTCs detection represents a promising perspective.

In this regard, we have recently set up a European project (InveNNta) in collaboration with the Iberian Nanotechnology Laboratory (INL, Braga, Portugal) that combines nanotechnology with real-time biosensing detection. In this project, a magnetoresistive biochip that detects magnetically tagged targets [107] will be adapted for CTCs counting. This technology has been successfully optimised for different biomedical applications [108,109] and represents a promising tool for CTCs research progress.

It is important to remark that introduction of biosensors for CTCs analysis could also help to circumvent one of the main challenges in the field, the possibility of expanding these cells *in vitro* due to a better viability preservation. This would also permit a better characterisation of CTCs from patients, which could, for example, lead to progress in individualised anti-tumour therapies. Although *in vitro* CTCs culture remains difficult to achieve, some promising results are emerging [110], supporting the idea of a new era in the oncology field.

## 5. Conclusions

CTCs isolation and analysis is one of the most challenging areas for translational cancer research. They are considered tumour liquid biopsies providing information of great value about the prognosis and prediction of therapy response. Several approaches have been developed in recent years to improve the efficiency of CTCs isolation and reduce the time needed for subsequent analysis. These technologies have the aims of cancer cell detection, single cell sensitivity, high selectivity, high reproducibility, easy fabrication, and low cost. The application of biosensors for CTCs analysis could cover these requirements and the few studies conducted to date have demonstrated promising results. However, biosensor technology for CTCs quantification needs further development to be incorporated into cancer management in terms of a commercial product.

## Figures and Tables

**Table 1.** Methods for CTCs isolation.

**CTCs Isolation**			**CTCs Detection**	**References**
IMMUNOAFFYNITY

	CELLSEARCH	anti-EpCAM	CD45; CK	[9,22–26]

	IMAGESTREAM	anti-EpCAM; flow cytometry	CD45; CK	[27]

	MULTICOLOUR FLOW CYTOMETRY	anti-CD45-; flow cytometry	CD45; EpCAM; CK	[28]

	MAGNETIC SIFTER	anti-EpCAM	CD45; EpCAM; CK; EGFR mutation	[29,30]

	GILUPI	anti-EpCAM	CD45; EpCAM; CK	[31]

	ISOLATION OF EPITHELIAL TUMOUR CELLS: ISET	Size	CK; PSA	[32–34]

PHYSICAL PROPERTIES
	SCREENCELL	Size	CD45; CK	[35,36]

	MEMS	Size	not established	[37,38]

	ONCOQUICK	Density	CK	[39]

	CTC-CHIP	Microfluidics	CK;CD45; PSA	[40]

	HB-CHIP	Microfluidics	CK;CD45; PSA	[41–43]

	MICROFLUIDIC PLATFORM	Microfluidics	CD45; CK	[44,45]

	DIELECTROPHORETIC FIELD FLOW FRACTIONATION CHAMBER	Microfluidics	not established	[46–51]

	MULTI-ORIFICE FLOW FRACTIONATION DEVICE-MOFF	Microfluidics	CD45; EpCAM; CK	[52]

**Table 2.** Sensors for CTCs detection.

**Biosensor Principle**	**Subtype of Transductor**	**Limit of Detection**	**References**
APTASENSOR	Quantum dot label	5 fM of *CEA* fragments	[78]
APTASENSOR	Resonance frequency shifts	4 LNCap cells/10 mL of blood	[79]
ELECTROCHEMICAL	Amperometry	DU145 cells concentration of 125 cells per sensor	[80]
ELECTROCHEMICAL	Impedance	-	[81]
ELECTROCHEMICAL	Conductometry	10 MDA-MB-468 cells/mL of blood	[82]
ELECTROCHEMICAL	Conductometry	10 ± 1 MCF-7 cells/mL of blood	[42]
ELECTROCHEMICAL	Conductometry	-	[83]
MASS CHANGE	Ultrasound	-	[84]
OPTICAL	Reflectometric interference spectroscopy	1,000 PANC-1 cells/mL	[85]
